# Intermittent chronic telogen effluvium with an unusual dermoscopic finding following COVID‐19

**DOI:** 10.1002/ccr3.6228

**Published:** 2022-08-09

**Authors:** Nasrin Saki, Fatemeh Sari Aslani, Mozhdeh Sepaskhah, Mohadese Shafiei, Sara Alavizadeh, Seyed Ali Hosseini, Fatemeh Ansari Asl, Najmeh Ahramiyanpour

**Affiliations:** ^1^ Molecular Dermatology Research Center Shiraz University of Medical Sciences Shiraz Iran; ^2^ Department of Dermatology, School of Medicine Shiraz University of Medical Sciences Shiraz Iran; ^3^ Pathology Department Shiraz University of Medical Sciences Shiraz Iran; ^4^ Department of Dermatology, Afzalipour Hospital, Afzalipour Faculty of Medicine Kerman University of Medical Sciences Kerman Iran; ^5^ Postdoctoral Researcher Shiraz University of Medical Sciences Shiraz Iran

**Keywords:** COVID‐19, Dermoscopy, telogen effluvium

## Abstract

Various conditions, including infections, can cause telogen effluvium (TE). One of them is coronavirus disease 2019 (COVID‐19), where hair loss usually begins between 2 and 12 weeks after the illness. TE can be acute or chronic, and the chronic type can be intermittent. Here, we present the case of a 17‐year‐old girl with severe and widespread hair loss following an upper respiratory infection suspected to be COVID‐19, with the patient having a history of such attacks since childhood. Evidence from biopsy and dermoscopy indicated a diagnosis of TE.

## Graphical abstract

Post‐COVID‐19 telogen effluvium is a diagnosis of exclusion, managed by treating the underlying cause, reassuring the patient, and providing appropriate supplements. We reported a rare case of high‐intensity telogen effluvium episodes following even mild upper respiratory infections in an adolescent, and documented bayonet hair as an unprecedented dermoscopic finding.

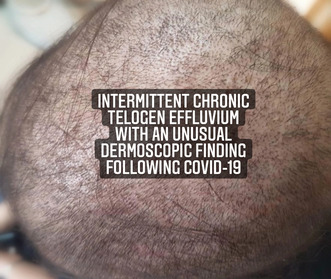

## 1 INTRODUCTION

Telogen effluvium (TE) is a disorder of the hair growth cycle. The proposed mechanisms are anagen phase shortening and early entry of hair into the telogen phase.^1^ Conditions that can cause TE include psychological stress, crash dieting, estrogen depletion, hypothyroidism, organ failure, trauma, medications, iron deficiency, severe infection, and acute febrile illnesses such as coronavirus disease 2019 (COVID‐19).^1^, ^2^, ^3^, ^4^

More than 25% of people with COVID‐19 experience TE, with women being affected more than men. The onset of TE is usually 2–12 weeks after COVID‐19, and patients with a more severe illness are more likely to develop TE.^4^ TE is generally divided into acute and chronic, often manifesting acutely.^1^, ^2^ The chronic type often appears intermittently and is associated with various stressors, including emotional stressors.^5^ Because it causes widespread hair loss, TE can lead to patient anxiety.^2^ Herein, we report a case of severe intermittent hair loss in a teenage girl following upper respiratory infection symptoms.

## 2 CASE PRESENTATION

**FIGURE 1:**
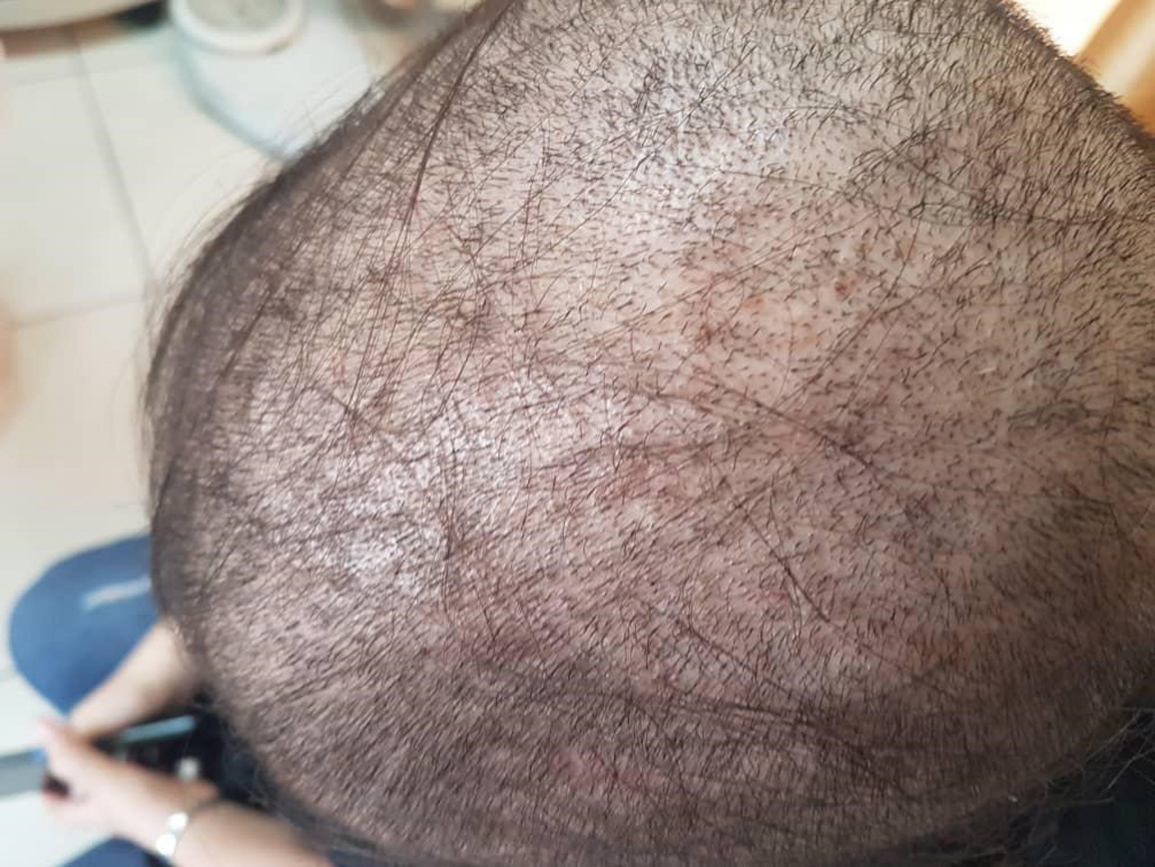
The patient's scalp on initial examination. Widespread hair loss is evident

A 17‐year‐old girl with no history of any specific diseases presented to our dermatology clinic with severe hair loss in September 2021. The hair pull test was strongly positive, and the fragility of the hair shaft was evident. As shown in Figure 1, hair loss was widespread. Two weeks earlier, the patient had a cough, rhinorrhea, and a sore throat. Her upper respiratory infection was treated on an outpatient basis with azithromycin 250 mg once daily and amoxicillin 250 mg every 8 hours. One week after the onset of symptoms, the patient's hair loss began. Interestingly, her parents mentioned that she had experienced a period of hair loss from childhood every time she had an upper respiratory infection, which would heal on its own. The parents also mentioned that in their view, this time, their daughter's respiratory infection was more severe than during her childhood, as was the rate of her hair loss. The timing of the infection (during a major COVID‐19 peak), the lack of vaccination against COVID‐19, and the positive recent history of documented COVID‐19 in close contacts led us to reasonably assume that the girl's infection was COVID‐19, though no laboratory confirmation was available.

**FIGURE 2:**
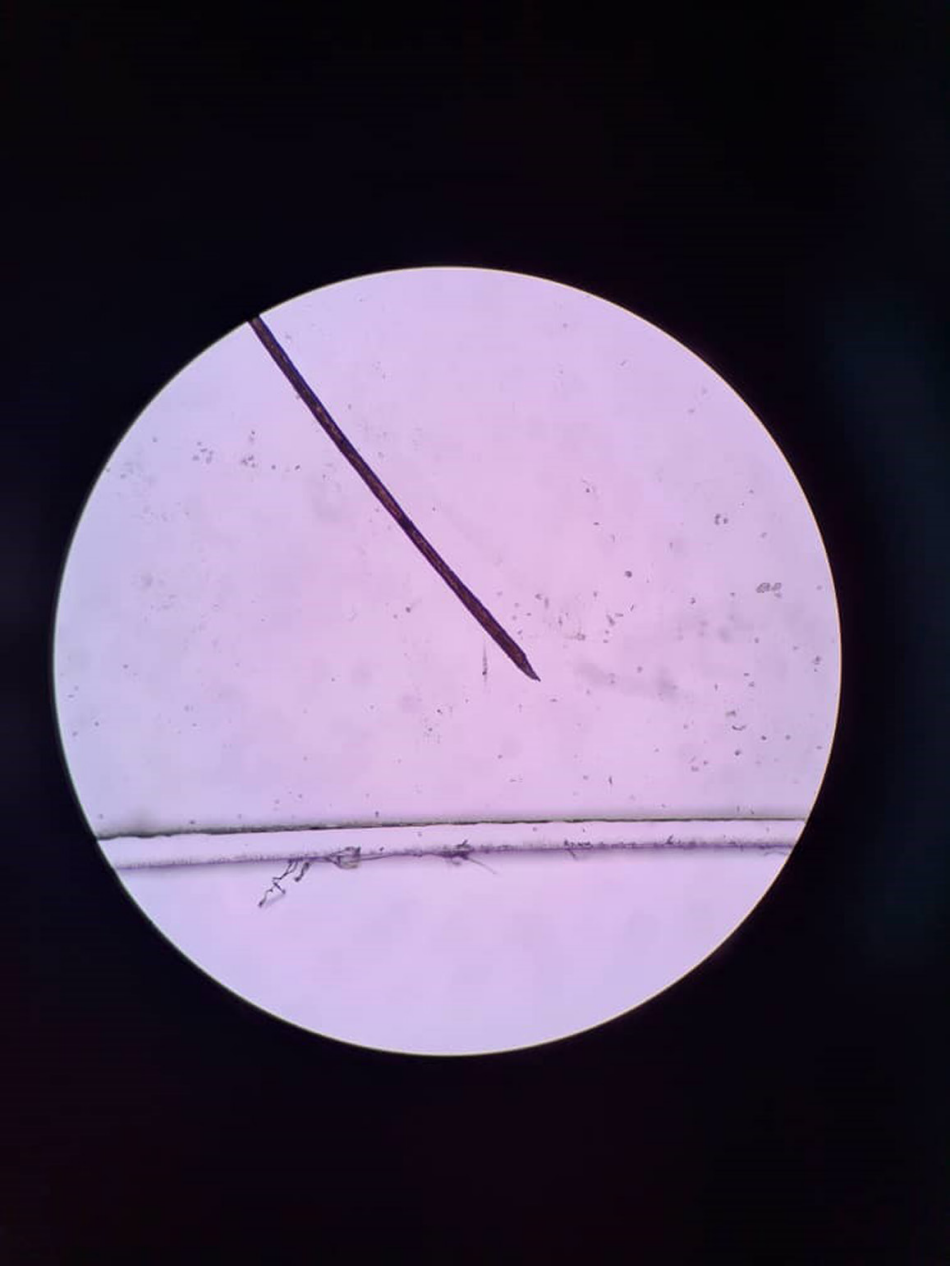
An image from the dermoscopy revealing a hair with an oblique tapered fracture

**FIGURE 3:**
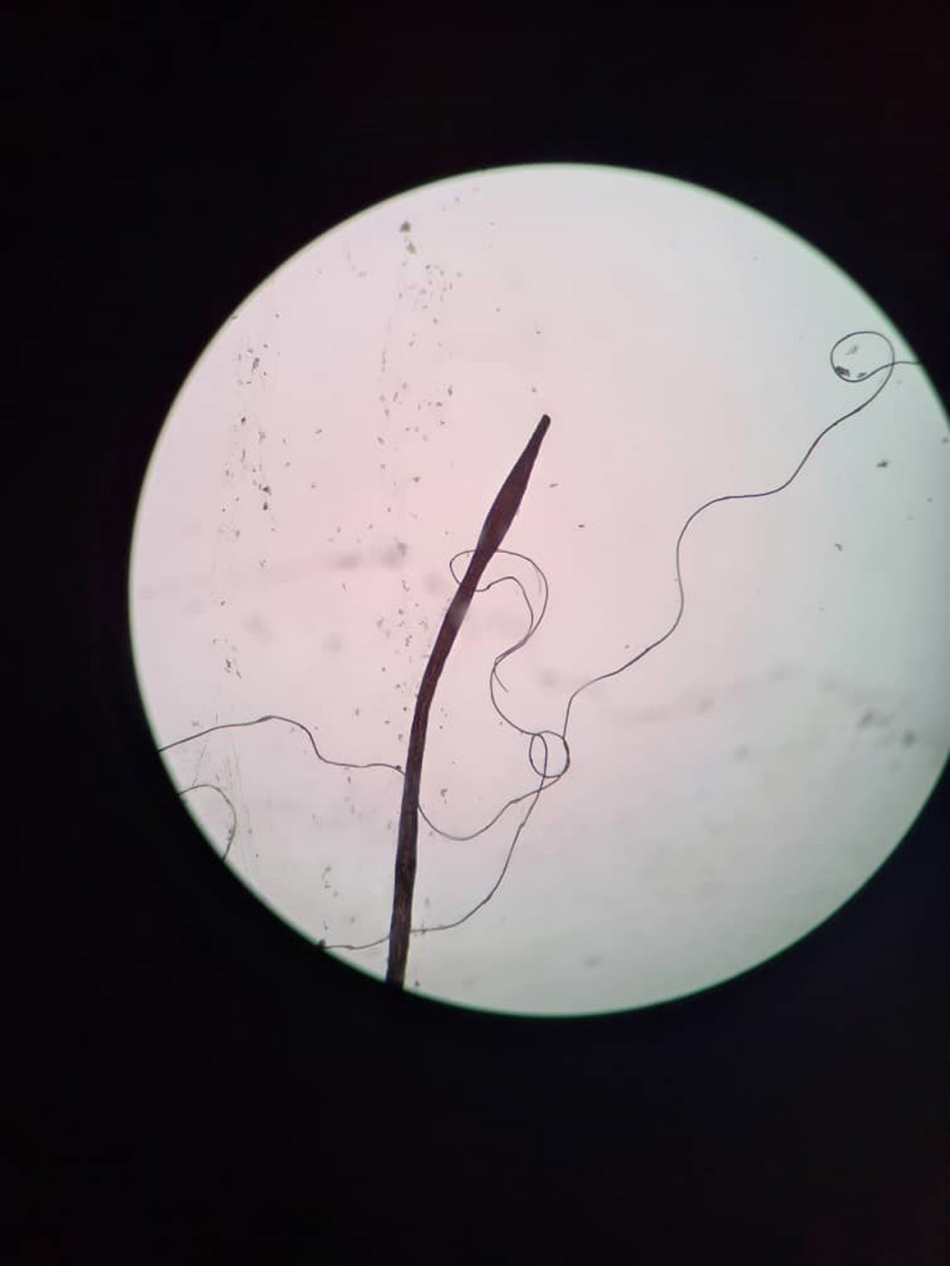
An image from the dermoscopy revealing a bayonet hair

We requested a dermoscopy and biopsy for the patient. The dermoscopy revealed normal hair thickness with empty follicles, indicative of TE. Notably, bayonet hair (Figure 2) and hairs suspicious for oblique tapered fracture (Figure 3) were also seen. The microscopic findings from the 4 mm scalp biopsies were as follows: Vertical sections showed hyperkeratosis with follicular plugging, moderate irregular acanthosis, and mild spongiosis. Dermis showed decreased hair follicle density, increased catagen/telogen follicles, and mild perivascular lymphocytic infiltration in the papillary dermis. No peribulbar inflammation, melanin casts, or fibrous tracts were seen. Periodic acid‐Schiff staining revealed many budding yeasts in the superficial keratin layer. Serial tangential sections showed increased catagen/telogen follicles and mild perivascular and mild focal perifollicular lymphocytic infiltration at the papillary dermal level. There also was an irregular border of hair shafts and abnormal hair shaft pigmentation. Periodic acid‐Schiff staining for fungus showed no fungal elements within the hair follicles. The conclusions of the 4 mm skin scalp punch biopsy (vertical and horizontal sectioning) were as follows: Nonscarring alopecia with increased catagen/telogen follicles associated with seborrheic dermatitis and abnormal hair shaft morphology. The final diagnosis was intermittent chronic TE.

**FIGURE 4:**
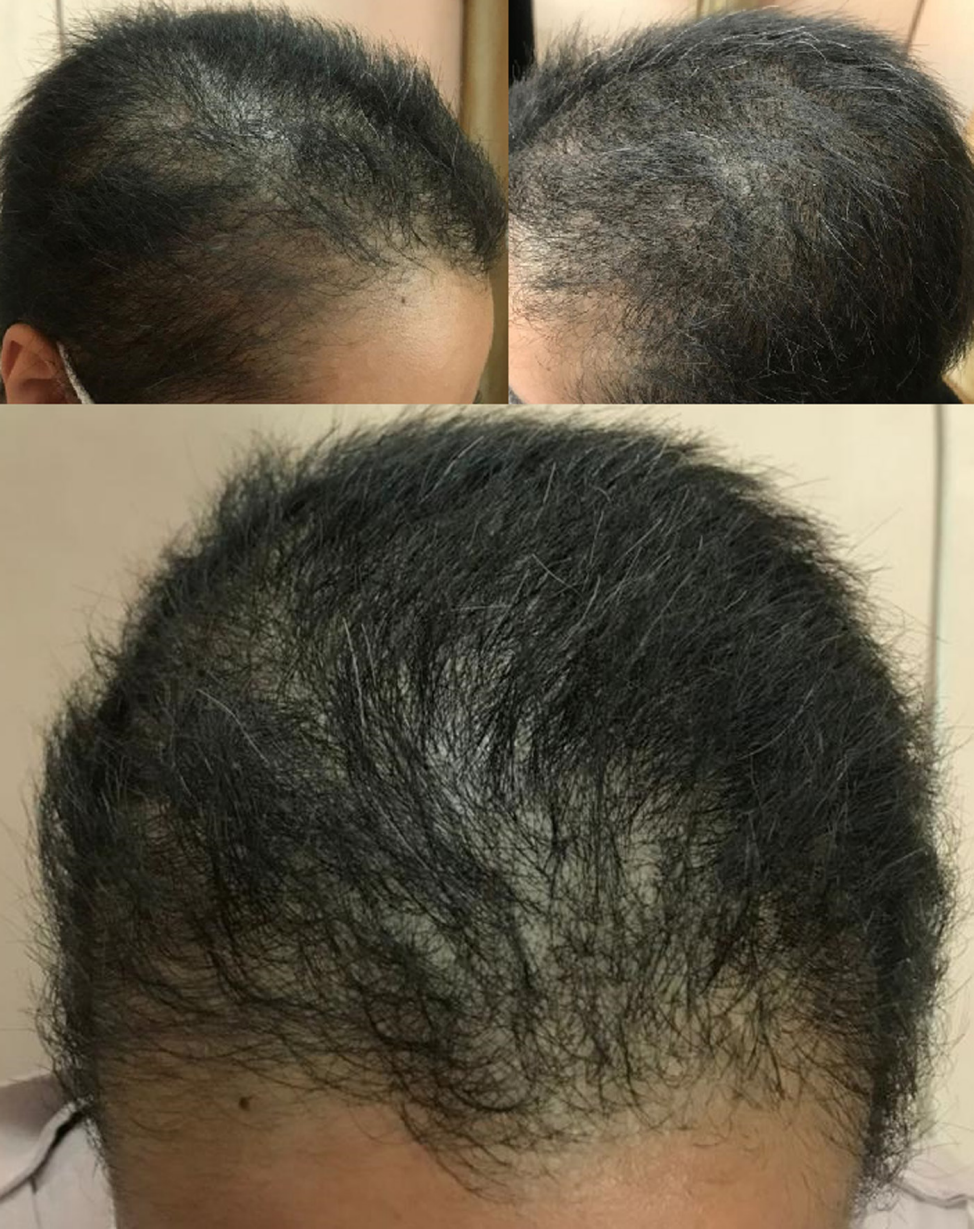
Partial recovery and hair growth after a month

We reassured the patient about the self‐limited nature of the condition and treated her with the following supplements: Vitamin D3 50,000 unit pearls weekly, biotin 5 mg intramuscular ampoules weekly, FolicoGen® tablets daily, and Cerita Minuta® herbal anti‐hair loss tonic, 30–60 drops applied to the hair every night. Partial recovery was achieved within a month (Figure 4).

## 3 DISCUSSION

Klingman described telogen effluvium (TE) for the first time in 1961^6^; it is a common cause of nonscarring hair loss, and its chief presentation is acute or chronic diffuse hair shedding. TE is much more common in women, usually affecting those aged 30–60.^7^, ^8^ Of course, women's sensitivity to signs of hair loss cannot be ignored when looking at this gender discrepancy.^9^

Post‐viral TE has become more common following the unprecedented COVID‐19 pandemic. Recent studies indicate an association between high levels of pro‐inflammatory cytokines (interleukin‐6, interleukin‐1β, interferon, and metalloproteinases 1 and 3) and acute TE.^10^, ^11^, ^12^ Furthermore, the activation of the coagulation cascade secondary to COVID‐19 decreases the concentration of anticoagulant proteins,^13^ which may induce the formation of microthrombi that occlude the blood supply of hair follicles.^14^ Direct infection of the hair follicle has also been implicated,^15^ with an early onset of TE suggesting this mechanism.^12^ Meanwhile, due to excessive consumption of drugs during the COVID‐19 pandemic, drug‐related TE cannot be overlooked. In fact, Waters et al. described the possible role of anticoagulants in inducing TE.^16^ On the other hand, emotional and psychological stress caused by the fear of COVID‐19 may also explain the increased prevalence of TE.^15^ Nonetheless, Aksoy et al. found that patients with and without COVID‐19‐associated TE were similar in terms of stress level and usage of COVID‐19 medications.^4^ Hence, the systemic inflammatory response and microthrombi formation represent the most likely mechanisms.^13^

In a study of 204 post‐COVID‐19 patients, the prevalence of COVID‐19‐associated TE was 27.9%. The condition manifested 2–12 weeks after COVID‐19 (average 53.76 days), with hypertension, the female sex, and hospitalization being more common in chronic TE patients.^4^ Strace et al. targeted post‐COVID‐19 patients referring to dermatology clinics due to skin or scalp diseases. They diagnosed TE in 66.3% of cases, and a significant portion of patients (62.5%) were recognized as early‐onset (<4 weeks) TE.^15^ In our case, the teenage girl appeared to have COVID‐19 roughly 2 weeks before the onset of this latest TE episode. In a study on changes in dermatologic disease profiles before and after COVID‐19, Kutlu et al. noted that the frequency of TE increased from 0.4 (May 2019) to 2.17 (May 2020), indicating a 5.5‐fold increase.^17^ Cline et al. reported that COVID‐19 was an essential trigger of TE, given a > 400% increase in TE patients since the pandemic commenced.^18^

Normal hair thickness with empty follicles, with or without hair thinning, are the usual features of dermoscopy in TE.^19^ In our case, which can be classified as intermittent chronic TE in a patient with diffuse hair shedding, we detected normal hair thickness and empty follicles on dermoscopy, with bayonet hair being documented as an unprecedented finding. Chemotherapy‐induced alopecia, alopecia areata due to malnutrition, and severe systemic infections can lead to intermittent fluctuations in keratinocyte proliferative activity, leading to contraction and expansion along the hair shaft. Eventually, these contractions break the hair shaft, causing the Pohl‐Pinkus sign (similar to an exclamation mark) to appear upon dermoscopy.^20^, ^21^, ^22^ One type of Pohl‐Pinkus mark is bayonet hair: a rare anomaly caused by damage to the anagen matrix of the hair, first named by Felix Pinkus.^23^ Following this injury, the distal hair shaft becomes dystrophic; as the shaft narrows, the human hair breaks at its weakest point and a spindle‐shaped appearance forms at the end of its cone.^24^, ^25^ Bayonet hair has been reported on dermoscopy in alopecia induced by chemotherapy, hypothyroidism, radiation and thallium poising.^25^, ^26^, ^27^, ^28^

The differential diagnosis of TE includes alopecia areata, androgenic alopecia and organic causes such as malnutrition, thyroid disorders and iron deficiency anemia.^19^ Given that TE can occur secondary to a wide range of conditions or can be mistaken with other diseases, the cornerstone of management is ruling out other conditions, treating the underlying cause, reassuring the patient that the disease is self‐limited and providing appropriate supplements. In similar cases, patients used topical minoxidil and corticosteroids, supplements containing amino acids (such as biotin), and vitamin B products.^14^, ^15^, ^18^, ^25^ We achieved an exact diagnosis, reassured the patient and treated her with vitamin D3, biotin, FolicoGen® and Cerita Minuta® herbal anti‐hair loss tonic, leading to partial recovery within a month.

Dermatologic manifestations following COVID‐19 in patients of different ages include a wide range of usual to unusual manifestations. According to a recent systematic review, patients with post‐COVID‐19 TE have a median age of 44 years, with the predominant dermoscopic findings being decreased hair density, empty follicles and short regrowing hair.^11^ The age of our patient deviated considerably from this typical disease picture. While our patient did have empty follicles on dermoscopy, the unprecedented finding of bayonet hair was documented, along with oblique tapered hair fractures. Another notable aspect was the intermittent and severe pattern of hair loss with remission and relapse periods, where even mild upper respiratory infections, including COVID‐19, appeared to be the main trigger. Repetition of alopecia attacks with this intensity and extent after any even mild upper respiratory infection is rare and may indicate a genetic predisposition. If similar cases are reported, genetic studies are recommended to check the possibility of a specific gene being associated with this condition.

Our aim in this study was to showcase the clinical and laboratory manifestations of TE following an upper respiratory infection, facilitating early diagnosis and treatment. Due to this condition's enormous psychological and economic burden on patients, targeted studies are required to understand its exact pathogenic mechanisms, and physicians should maintain their knowledge up to date as the COVID‐19 pandemic continues.

## AUTHOR CONTRIBUTIONS

NS and SA and FA: Involve Follow Up and Visit. MS and FS: Pathology Diagnosis. AH and MSH and NA: Data Collection and Manuscript Drafting. NS and NA: Final Manuscript.

## CONFLICT OF INTEREST

The authors declare no conflicts of interest.

## CONSENT

Published with the written consent of the patient.

## Data Availability

Data are available upon reasonable request to the corresponding author.
